# Evaluating the neonatal BCG vaccination programme in Ireland

**DOI:** 10.1186/s13690-016-0141-0

**Published:** 2016-07-13

**Authors:** Cara Usher, Roisin Adams, Susanne Schmitz, Jennifer Kieran, Darina O’Flanagan, Joan O’Donnell, Kevin Connolly, Brenda Corcoran, Karina Butler, Michael Barry, Cathal Walsh

**Affiliations:** National Centre for Pharmacoeconomics, St. James’s Hospital, Dublin 8, Ireland; Department of Pharmacology & Therapeutics, Trinity Centre, St. James’s Hospital, Dublin 8, Ireland; Department of Maths & Statistics, Centre for Health Decision Sciences (CHeDS), University of Limerick, Limerick, Ireland; Health Protection Surveillance Centre, Dublin 1, Ireland; National Immunisation Advisory Committee, Dublin 2, Ireland; National Immunisation Office, Dublin 7, Ireland; Department of Infectious Diseases, Our Lady’s Childrens Hospital, Crumlin, Dublin 12, Ireland

**Keywords:** BCG vaccine, Paediatric tuberculosis, Vaccine efficacy, Cost effectiveness, Neonatal vaccination

## Abstract

**Background:**

The aim of this study was to compare the cost effectiveness of the current Irish programme of universal BCG vaccination of infants versus a programme which considered selectively vaccinating high risk infants using decision analytical modelling.

**Methods:**

The efficacy of the BCG vaccine was re-evaluated to inform a decision analytical model constructed to follow a birth cohort of vaccinated and unvaccinated infants over a 15 year time horizon. The number of life years gained (LYG) was the primary outcome measure and this was compared to the net cost of the vaccination strategies.

**Results:**

In the base case analysis, the incremental cost effectiveness ratios (ICERs) for the universal strategy and selective strategy vs no vaccination were €204,373/LYG and €143,233/LYG respectively. When comparing the incremental difference in moving from the universal to the selective strategy, the selective strategy costs €1,055,692 less per 4.8 life years lost per birth cohort. One way sensitivity analyses highlighted that a move from the universal to the selective strategy was particularly sensitive to the estimate of vaccine efficacy against deaths, the cost of administering the vaccine and the multiplier used to apportion risk of contracting tuberculosis. Probabilistic analysis suggested that a move from a universal based strategy to a selective based strategy could be deemed cost effective (probability of cost effectiveness is 76.8 %).

**Conclusion:**

The results of the study support the protective effect of the BCG vaccine in infants and quantified the cost effectiveness of the current BCG vaccination strategy and the decremental difference in moving to a selective strategy. This analysis highlights that the additional protection offered by the universal vaccination strategy is small compared to that of the selective strategy. Consideration should therefore be given to the implementation of a selective vaccination strategy, and diverting resources to improve TB case management and control.

**Electronic supplementary material:**

The online version of this article (doi:10.1186/s13690-016-0141-0) contains supplementary material, which is available to authorized users.

## Background

As the incidence of TB continues to decline in high and middle income countries and because of the conflicting data on its protective efficacy, a valid and pressing question is whether BCG vaccination should be discontinued or targeted at certain groups, known to have a higher risk of contracting infection. The International Union Against Tuberculosis and Lung Diseases (IUATLD) recommends that routine vaccination be discontinued when the average annual notification rate of sputum smear positive pulmonary TB is 5 per 100,000 population or less during the previous 3 years [1].

As well as the IUATLD criteria, there are additional considerations, such as economic analysis and societal preference, which should be addressed when deciding to modify or stop a universal BCG programme. Ireland and Portugal remain the only countries in Western Europe implementing universal BCG vaccination programmes. France revised its approach to the use of the BCG vaccine, firstly in 2004, when revaccination with BCG ceased [2] and then in 2007 when routine vaccination of all school children ceased and a more targeted approach to vaccination was introduced [3]. The national policy in Finland was changed in 2005, whereby the universal vaccination policy was changed to a targeted approach of high risk infants [4]. Likewise in the UK, a review and revision of the BCG vaccination policy lead to the implementation of a selective vaccination strategy of high risk groups in favour of the universal schools programme which had been in place since the 1950s. Some countries with low rates of TB, such as Sweden and Switzerland, discontinued their universal BCG vaccination strategies many years ago [5]. Japan has also reappraised the value of routine BCG vaccination in the prevention of TB [6]. These policy differences are mainly related to differences in opinion about the efficacy of the vaccine and local variations in TB epidemiology.

In 2012 the National Immunisation Advisory Committee (NIAC) commenced an economic analysis of the existing universal BCG vaccine programme in Ireland. The aim of the assessment was to examine the cost effectiveness of the current BCG vaccination programme of infants using revised estimates of vaccine efficacy and to quantify the incremental difference in moving to a selective based strategy, which identified and vaccinated high risk infants only. The process and results of this assessment are presented.

## Methods

### Framework

Prior to commencing the evaluation, the scope of the analysis was agreed with an expert advisory group. The base-case parameters for the model were established and the most appropriate data inputs were collected for the model.

### Perspective

The analysis was undertaken from the perspective of the Health Service Executive (HSE), i.e., the healthcare payer. Therefore only direct medical costs were included in the evaluation. Costs associated with productivity changes due to parental time off work were included in a separate scenario analysis.

### Vaccination strategies

For the universal BCG strategy, it is assumed that a birth cohort of infants (*n* = 72,410) is vaccinated with a coverage rate of 80 % (of the birth cohort). The size of the birth cohort in this analysis (*n* = 72,410) is based on the population estimates in Ireland for 2011.

For the selective strategy, the target group of high-risk children was estimated to be 11.7 % of the birth cohort with a coverage rate of 44 %. The definition of “high-risk’” was infants with at least one parent from a high TB incidence country (≥ 40 cases per 100,000 persons) and was based on information taken from the “Growing up in Ireland” study which showed that 10.7 % of fathers and 12.7 % of mothers of the infant cohort (born between December 2007 and May 2008) were born in Eastern Europe, Africa and the “Rest of the World”-regions which could be considered areas of high TB endemnicity [7].

The children in the high risk group are assumed also to have a risk of TB which is three times (x3) higher than the 88.3 % of children in the remaining low-risk group. This assumption is based data provided in the HPSC Report on the Epidemiology of Tuberculosis [8]. The overall incidence is a weighted average between the two groups, and thus the increased risk in the high risk group is matched by a decreased risk in the remainder of the population.

It is assumed that the vaccine would be administered to the neonates before they reach 3 months of age and would be delivered in the community setting.

### Model structure

A decision analytic model was constructed to follow a birth cohort of vaccinated and unvaccinated individuals from birth over a 15 year period. The model is based on a series of health states (i.e., pulmonary TB, extrapulmonary TB, meningeal TB and death) that an individual can occupy at a given point in time and it is run in annual cycles. The model estimates the number of cases of pulmonary TB, extrapulmonary TB and TB meningitis averted in the birth cohort of 72,410 infants. Age-specific background mortality was based on life tables from the Irish Central Statistics Office [9]. The number of life years gained (LYG) from the vaccination programmes was the primary health outcome measure, and these were compared to the net cost i.e., the additional cost of universal or selective vaccination minus the expected savings from reduced use of healthcare resources, due to a reduction in the burden of TB disease. The model was fitted with local cost, resource use and epidemiological data.

In the evaluation, cases of pulmonary TB, extrapulmonary TB, and meningeal TB are always initially managed as inpatients, and this reflects current clinical practice in Ireland. TB meningitis can lead to long term complications (hearing loss, focal neurological deficits, development delay, epilepsy) which are also incorporated into the analysis. The analysis was performed in MS Excel 2010.

### Comparator

The vaccination strategies were compared to no vaccination, i.e., BCG_universal_ vs BCG_no vaccination_ and BCG_selective_ vs BCG_no vaccination,_ before comparing the incremental difference in moving from a universal strategy to a selective strategy.

## Model inputs

### Vaccine efficacy

Several systematic reviews to date conclude that BCG vaccination of infants is very effective in preventing miliary TB and TB meningitis in children [10]. One meta-analysis conducted in the USA [11] provided evidence for a protective effect of the BCG vaccine against pulmonary TB, TB deaths, TB meningitis, laboratory confirmed TB cases and disseminated TB. In 1995 Colditz and colleagues (13) reviewed the results of 5 randomised control trials and 11 case control studies. They estimated the protective effect of the BCG vaccine for preventing pulmonary TB, TB deaths, TB meningitis, laboratory confirmed TB cases and disseminated TB. Trunz and colleagues [12] re-evaluated BCG efficacy against childhood TB meningitis and miliary TB by adding seven more published investigations to earlier meta-analyses of published case-control studies [10, 11]. A total of 18 case-control studies provided revised estimates of efficacy for TB meningitis and miliary TB, which are similar to earlier published estimates [10, 11, 13].

**Fig. 1:**
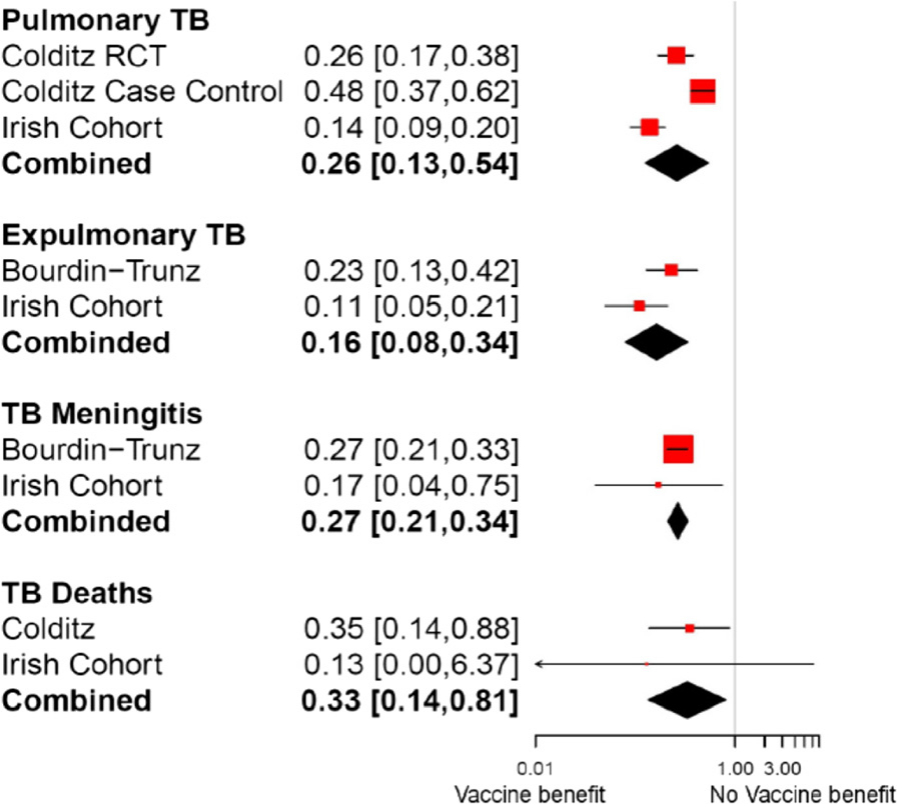
Estimates of vaccine efficacy from meta-analysis and combined results used in the model. Shown are risk ratios and 95 % confidence intervals. The size of the red squares indicates the relative influence of the trial on the combined estimate; trials of large sample size will have a higher impact compared to trials of smaller sample size. The black diamond illustrates the combined estimate for each outcome; its width is a measure of the associated uncertainty

In Ireland, the Health Protection Surveillance Centre (HPSC) documents all cases of notified TB. In addition to documenting cases, other information such as age, gender, local health office/county, country of birth, BCG vaccination status, presence of BCG scar, and treatment outcome is recorded. It therefore serves as a reliable source to estimate the effectiveness of the BCG vaccination in the Irish population. To this end, a retrospective evaluation of TB cases, stratified according to BCG status from the years 2002 to 2011 was conducted. The analysis was fitted in R (version 2.15.2) to combine this Irish data with data from Colditz [11] and Trunz [12]. Resulting risk ratios provide estimates of vaccine efficacy for the Irish population aged 0-15 years strengthened by international estimates. A forest plot of the results is shown (Fig. 1), illustrating the impact different data sources have on the revised combined estimates.

### Vaccine uptake

Not all local health offices report their BCG vaccine uptake rates to the HPSC. The mode of delivery also varies across the country with approximately 75 % of all BCG vaccinations being performed in local health offices and the remainder (25 %) being done in maternity hospitals (National Immunisation Office (NIO), personal communication). For the purposes of the current study, it was assumed that the vaccine uptake in the base case analysis would be approximately 80 % for the universal strategy and was varied by 75–95 % in the sensitivity analysis. These figures were based on expert clinical opinion (from the NIO) and the latest HPSC immunization statistics [14].

For the selective strategy, it was assumed that the vaccine uptake in the base case analysis would be approximately 44 % and was varied by 38–50 % in the sensitivity analysis. These figures were based on experience from France [15], where, following a change in their vaccination schedule to a selective based strategy, reported uptake rates of lower magnitude than those experienced for the universal strategy. Experience from other low incidence countries, showed similar evidence for a reduced uptake rate for a selective based strategy, e.g., Sweden reported a vaccine uptake rate of less than 2 % in 1976 a year after changing to a selective based strategy [5].

### Incidence of childhood TB

In the southern area of Ireland, a universal neonatal BCG vaccination was offered in Cork up to 1972 when a decision was taken to discontinue the vaccine for a variety of reasons. This small cohort, together with the information provided by the HPSC, therefore provided a reliable source to estimate the incidence of childhood TB in a non-vaccinated Irish population. Annualized incidence rates were calculated for each TB health state, for each year between 0 and 15 years, and these were used as the basis to build the model. In a separate scenario analysis, which examined the effect of the vaccine up to 25 years, annualized incidence rates from those aged 16–25 years were used (Table 1).

**Table 1:** Annualised incidence rate of TB clinical syndromes and mortality by age in a non-vaccinated cohort of Irish children 2002-2010

Age group	Annualised incidence rate of Pulmonary TB per 1000 population	Annualised incidence rate of Extrapulmonary TB per 1000 population	Annualised incidence rate of TB Meningitis per 1000 population
Under 1 year	0.015	0.000	0.015
1 year	0.000	0.015	0.000
2 years	0.196	0.075	0.000
3 years	0.133	0.015	0.000
4 years	0.030	0.015	0.000
5 years	0.031	0.015	0.000
6 years	0.016	0.000	0.000
7 years	0.000	0.015	0.015
8 years	0.031	0.015	0.000
9 years	0.031	0.000	0.000
10 years	0.049	0.032	0.000
11 years	0.032	0.016	0.000
12 years	0.017	0.033	0.017
13 years	0.032	0.000	0.000
14 years	0.063	0.031	0.000
15 years	0.061	0.000	0.000
16 years	0.031	0.031	0.000
17 years	0.047	0.032	0.000
18 years	0.074	0.044	0.000
19 years	0.056	0.028	0.000
20 years	0.053	0.040	0.000
21 years	0.130	0.026	0.000
22 years	0.079	0.039	0.000
23 years	0.103	0.039	0.013
24 years	0.075	0.000	0.000
25 years	0.108	0.060	0.012

### Mortality data

There were no recorded deaths due to TB in the 0–15 year old population, so a case fatality rate (CFR) of 0.8 % (based on UK Surveillance data from 2001 to 2010, compiled by the Health Protection Agency, UK [16]) was applied.

### Long term complications of TB meningitis

There is a wide range of possible long-term sequelae following meningitis and the analysis is restricted to four main complications: hearing loss, developmental delay, focal neurological deficits andepilepsy. The probability of these events occurring was derived from published studies.

In 1964, Todd and Neville [17] estimated the incidence of hearing loss and epilepsy attacks. They combined the results of their own study with those of Wasz-Hockert & Donner [18], Lorber [19], Voljavec et al. [20], Pohitonova [21] and Lapides [22]. From these 6 studies they combined information on the incidence of sequelae in 855 children who had survived an attack of meningeal TB. The estimates for hearing loss and epilepsy attacks were 7 and 7.6 % respectively. Lorber [19] estimated the incidence of cognitive impairment at 50 %. Schoeman [23] estimated that focal neurological deficits occurred in 25 % of cases analysed. The probabilities of long-term complications of meningitis were varied by +/−20 % in the sensitivity analysis.

### Resource use data

#### Cost estimates

Cost estimates are explained in terms of healthcare resource use (i.e., vaccination costs and direct medical costs) and unit cost data.

### Vaccination costs

Vaccination cost estimates were obtained from the NIO of the HSE. The cost of vaccination includes the cost of the vaccine as well as the cost of administration of the vaccine. The vaccine ingredient costs are calculated as 80 or 50 % of the cost of distributed vaccine in 2012 for the universal and selective strategies respectively (Personal communication with NIO). The cost of administration is based on an administration fee of €27.75 and €50 per infant, for the universal strategy and selective strategies respectively. The higher fee for the selective strategy reflects the difficulty that may be present in identifying those at high risk. Changes to the administration fee are explored in the sensitivity analysis.

### Direct medical costs

Due to the lack of published Irish cost data, and the time constraints with conducting specific micro-costing studies, an assessment of resource use items associated with diagnosis and treatment of each of the health states (pulmonary TB, extrapulmonary TB and TB meningitis) were obtained by the evaluation team with local clinical experts. The costs associated with contact tracing per primary case of TB and diagnosing and treating 50 % of those contacts for latent TB were added to the direct medical costs of pulmonary TB, extrapulmonary TB and TB meningitis. Costs of contact tracing and latent TB are based on 9.4 contacts per TB case and are shown in Table 2. The costs of managing the long-term sequelae of meningitis were also included and the overall cost shown in Table 2. A comprehensive list of resource utilisation and unit cost data used to estimate direct medical costs of latent TB, pulmonary TB, extrapulmonary TB, TB meningitis and long term sequelae of meningitis are provided in Additional files 1, 2, 3, 4 and 5 respectively.

**Table 2:** Summary of parameter estimates, with base case values, range, distributions and sources

Parameter	Base case estimate	Range for one-way sensitivity analysis	Probability distribution for PSA	Source
Incidence of TB: see Table 1.				
Vaccine efficacy	Probability			
Pulmonary TB	0.74	0.45–0.87	Beta	[11, 12] Irish HPSC data 2002–2011
Extrapulmonary TB	0.84	0.66–0.92	Beta	
TB Meningitis	0.73	0.66–0.79	Beta	
TB Deaths	0.67	0.19–0.86	Beta	
Scenario Analysis: 0–25 year old cohort				
Vaccine Efficacy (against TB)	0.55	0.31–0.77	Beta	[39]
Vaccine Efficacy (against TB death)	0.44	−0.22–0.75	Beta	[39]
Direct costs- children (average cost per acute episode per child)				
Pulmonary TB	€8,153	+/−20 %	Log Normal	NCPE 2012
Extrapulmonary TB	€12,223	+/−20 %	Log Normal	NCPE 2012
TB Meningitis	€15,752	+/−20 %	Log Normal	NCPE 2012
Contact tracing per primary case of TB	€4,248	+/−20 %	Log Normal	HSE East 2012
Latent TB per primary case of TB	€4,203	+/−20 %	Log Normal	NCPE 2012
Long term costs due to meningitis (over 15 years)	€36,225.39	+/−20 %	Log Normal	NCPE 2012
Vaccine Ingredient costs (per strategy)				
Universal (80 % of the cost of distributed vaccine in 2012)	€155,215	+/−20 %	Fixed	National Immunisation Office / HSE procurement
Selective (50 % of the cost of distributed vaccine in 2012)	€97,010			
Administration cost per dose				National Immunisation Office / HSE procurement
Universal	€27.75 per dose		Point	
Selective	€50 per dose	+/−50 %		
Vaccine coverage			Uniform	National Immunisation Office [14, 15]
Universal	80 %	79–97 %		
Selective	44 %	38–50 %		
Case Fatality Rate (CFR)	0.8 %	0.5–1.1 %	Beta	[16]
Discount rate for costs and benefits	4 %	0–6 %	Point	[38]
Incidence of adverse events	1/1000	> 1/10,000 to < 1/100	Beta multiplier	[25]
Costs per treatment for adverse event	€2,895	+/−20 %	Log Normal	NCPE 2012
Multiplier used for high risk population	3-fold	x 2–x 8	Uniform (on log scale)	[8]
Proportion of birth cohort estimated to be at high risk	11.7 %	10.7–12.7 %	Uniform	[7]
Indirect Costs Value of work loss/week for parent of children with an adverse event or TB		€476.40		[40]
Percentage of parents taking time of work	Not included	42 %	Not included	[41]
Number of days of work lost		4		

*NCPE* National Centre for Pharmacoeconomics, *HSE East*: Health Service Executive East

### Unit cost data

Unit costs for inpatient procedures were obtained from 2010 Diagnosis Related Group (DRG) data provided by the National Casemix Unit of the HSE [24]. Unit costs for tuberculin skin tests were obtained from the Finance department of a local university teaching hospital (St. James’s Hospital, Dublin). Costs of antimicrobial therapy were obtained from the Pharmacy department of St. James’s Hospital Dublin. Every effort was made to incorporate Irish unit cost data. However, where data were not available it was adapted from the UK. UK costs were converted to euro using the exchange rate (€1.18) published by the Central Bank of Ireland and all costs were inflated to 2012 euro, using the consumer price index for health inflationary unit (105.6).

### Adverse events

The model uses the incidence rate of severe adverse events (1 in 1000) reported in the summary of product characteristics for the BCG vaccine [25]. Following review of all local reports [26–33] as well as international reports [34–36] published in the literature, none of the reports indicate that the rate is higher than this. The estimate of 1 in 1000 is applied only in the first year for the cohort as the literature indicates that the median onset of adverse effects ranges from 30 days to 4 months [32–35]. This rate is varied in the sensitivity analysis from > 1/10,000 to < 1/100 which is within the rates reported in the summary of product characteristics [25].

### Time horizon

The analytic time frame of the study was fifteen years in the base case analysis given the current paucity of data on the duration of vaccine protection beyond this time [37].

### Outcome measure

The main outcome measure in the analysis was cost per LYG.

### Discounting

An annual discount rate of 5 % was applied to both costs and consequences in the economic model, consistent with Guidelines for the Conduct of Economic Evaluations in Ireland [38].

### Sensitivity analyses

#### One-way

One-way sensitivity analysis was undertaken for the base case analysis. Ranges for the parameter values used are shown in Table 2.

### Probabilistic sensitivity analysis

A probabilistic sensitivity analysis was also conducted whereby all parameters were varied simultaneously. One thousand simulated combinations of the parameters were drawn and for each of these combinations a cost per LYG was estimated.

### Scenario analyses

#### Long term effect of vaccination

- A scenario analysis was conducted which examined the effect of vaccination up to 25 years. To do this, incidence data was gathered from those aged 16 to 25 years from non-vaccinated and vaccinated cohorts respectively and rates of vaccine efficacy against the TB health states (Protective effect: 0.55 95 % CI 0.31 to 0.77) and against TB death (Protective effect: 0.44 95 % CI−0.22 to 0.70) were applied [39].

#### Vaccinating a birth cohort-the societal perspective

- A scenario analysis was conducted whereby the indirect costs due to TB were included in the analysis. This analysis included the value of work lost per week [40] for a parent of a child with TB or an adverse event due to vaccination. This was based on the work of Roberts et al., [41], which stated that 42 % of parents would take time off work and an assumption that the number of work days lost is four.

## Results

### Baseline costs and effects

In the base case scenario, the model estimates the number of cases of pulmonary TB, extrapulmonary TB and TB meningitis averted in a birth cohort of 72,410 infants under the three scenarios (Table 3).

**Table 3:** Estimated number of cases (cases averted) in the three strategies over 15 years

Outcomes	BCG_universal (cases averted)_	BCG_selective (cases averted)_	BCG_no vacc_
Pulmonary TB	21.6 (31.4)	47.1 (5.9)	53.0
Extrapulmonary TB	6.6 (13.5)	17.6 (2.5)	20.1
TB meningitis	1.4 (1.9)	2.9 (0.4)	3.3
Total	29.6 (46.8)	67.6 (8.8)	76.4

For the universal strategy, the total cost of the BCG vaccine, including an administration fee of €27.75 per dose, would be approximately €1.7 million per year for a birth cohort of 72,410 infants. For the selective strategy, the total cost of the BCG vaccine, including an administration fee of €50 per dose, would be approximately €280,207 per year. The vaccine ingredient costs are calculated as 80 or 50 % of the cost of distributed vaccine in 2012 for the universal and selective strategies respectively.

The cost of diagnosing and treating TB disease was estimated to decrease by €848,199 and €160,945 in the universal and selective vaccinated cohorts respectively due to cost savings from avoided TB disease among the vaccinated cohorts (Table 4).

**Table 4:** Estimated direct costs (costs avoided) in the vaccination strategies

Direct treatment costs (avoided)	BCG_universal_ (costs avoided)	BCG_selective_ (costs avoided)	BCG_no vaccination_
Pulmonary TB	€359,205 (€521,200)	€781,507 (€98,898)	€880,405
Extrapulmonary TB	€136,539 (€279,738)	€363,197 (€53,080)	€416,277
TB meningitis	€33,666 (€47,262)	€71,960 (€8,968)	€80,928
Total	€529,410 (€848,199)	€1,216,664 (€160,945)	€1,377,609

The costs of treating adverse events due to vaccination were also estimated at €134,161 and €10,607 for the universal and selective strategies respectively.

#### Baseline incremental cost-effectiveness ratios

The base case ICERs, when comparing the universal and selective vaccination strategies to not vaccinating are €204,373/LYG and €143,233/LYG respectively. When comparing the incremental difference in moving from the universal to the selective strategy, the selective strategy costs €1,055,692 less per 4.8 life years lost per birth cohort (Table 5).

**Table 5:** Baseline incremental cost effectiveness ratios of the three vaccination strategies

	Costs	Life Years	∆ Costs	∆ Life Years Gained (LFG)	ICER (vs no vaccination)
No vaccination	€1,108,208	20110.0			
Selective	€1,270,131	20111.2	€161,924	1.1	€143,233
Universal	€2,325,823	20116.9	€1,217,616	5.9	€204,373
	Costs	Life Years	∆ Costs	∆ LYG	
Selective	€1,270,131	20111.2			
Universal	€2,325,823	20116.9	−€1,055,692	−4.8	Selective strategy less costly & less effective

### Sensitivity analyses

#### One-way

**Fig. 2:**
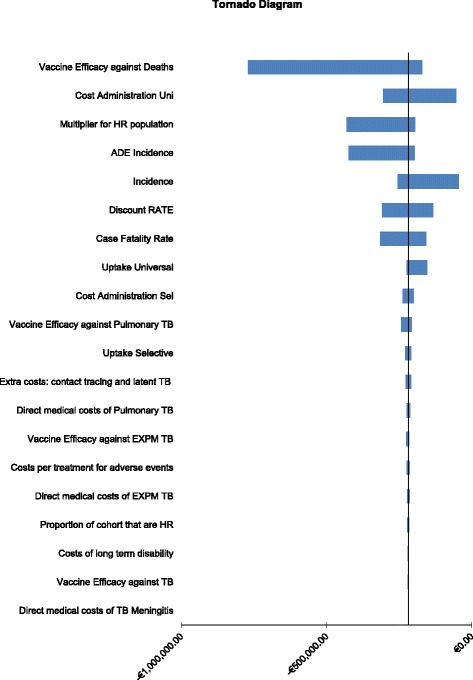
Tornado diagram depicting parameters of greatest influence in moving from a universal to a selective based strategy (Uni: universal, Sel: selective)

The main drivers of uncertainty in the model for the analysis of the incremental difference in going from a universal strategy to a selective strategy were as follows (Fig. 2);

- Vaccine efficacy against deaths
  When this parameter was varied by its 95 % CI estimates, it resulted in the selective strategy costing €1,055,692 less with 1.4 and 6.2 life years lost for the lower and upper CIs, respectively.
- Cost of vaccine administration
  When this parameter was varied by its 95 % CI estimates, it resulted in the selective strategy loosing 4.83 life years and costing €259,182 and €1,475,670 less for the lower and upper CIs, respectively.
- Multiplier
  When a risk of x2 is apportioned to the high risk population the selective strategy costs €1,012,728 less with a loss of 5.2 life years. When a risk of x8 is apportioned to the high risk population the selective strategy costs €1,270,508 less with a loss of 2.9 life years.

#### Probabilistic sensitivity analysis

**Fig. 3:**
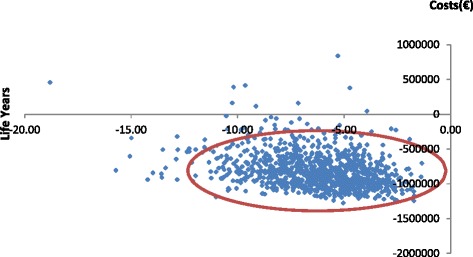
Incremental costs and LYG for the selective versus the universal vaccination strategy showing the 90 % confidence ellipse

A probabilistic analysis was run (1,000 simulations) and the incremental difference for selective versus universal is shown in Fig. 3 (i.e., universal at the origin) with a 90 % confidence ellipse depicted. The distribution of 1000 simulations shown in Fig. 3 shows 76.8 % of simulations occur below the willingness to accept threshold of at least €90,000/LYL in the South West quadrant.

### Scenario analysis

#### Long term effect of vaccination - Incremental cost-effectiveness ratios

When the model incorporates the effect of the vaccine on TB health states over 25 years, the ICERs for the universal and selective based strategies compared to not vaccinating are €167,878/LYG and €108,449/LYG respectively.

#### Vaccinating a birth cohort-the societal perspective

When the model incorporates the indirect costs of parents time off work, due to a TB related illness or a vaccine related adverse event, the ICERs for the universal and selective based strategies compared to not vaccinating are €204,966/LYG and €143,468/LYG respectively, which are comparable to the ICERs calculated in the base case analysis. A similar trend was observed to the base case analysis when moving from a universal to a selective based strategy.

## Discussion

The results of this economic evaluation suggest that both the universal BCG vaccination programme and the targeted strategy of selectively vaccinating high-risk neonates in Ireland would not be considered cost effective compared to not vaccinating. When compared to the universal programme, the targeted (or selective) strategy is substantially less expensive but demonstrates marginally less health gain (4.8 life years). Assuming however, that people demand twice the amount of money to relinquish one unit of health, compared to the amount they are willing to pay to gain an extra unit of health [42], the probabilistic analysis indicates that a move from a universal based strategy to a selective based strategy could be deemed acceptable.

While TB incidence has fallen dramatically in Western Europe and the US over the last century it remains a public health burden of global concern. Historically, Ireland has had high rates of TB compared with other countries in Western Europe but rates have been falling and it is now considered a low-burden country. However, public concern about TB remains. Results from our re-evaluation of vaccine efficacy confirm the protective effect of the vaccine in the local population, as demonstrated through studies conducted in Ireland in the late 80’s and early 90’s [43–52]. The addition of our estimates of vaccine efficacy to previously published systematic reviews and meta-analyses adds to the literature on the efficacy of BCG vaccine, an area which has been under-served and remains controversial. While decisions in this area have been largely taken in Europe, these questions remain to be considered in low resource countries with poor health infrastructures. The efficacy data presented, which combines data from a country with a previously high prevalence of TB to that of the international literature, will be a helpful and useful addition for economic analysis in those settings.

### Decrementally cost-effective medical interventions

With healthcare expenditures growing at a significant rate, decision makers struggle with reconciling their finite budgets with the reimbursement of innovative medical technologies, be they vaccines, pharmaceuticals or diagnostics. In such situations, cost-saving alternatives may become attractive, even if they come with reduced benefits. The theory is that limited benefits could be sacrificed for substantial resource savings, thus permitting reallocation to higher value alternatives. TB control is an area where this might be an appropriate approach, especially in low-burden countries. Targeting vaccinations to high-risk groups would allow re-allocation of resources into more robust TB screening and treatment programs. Less expensive, lower quality innovations are ubiquitous in other markets but have not been described in the health care sector to any great extent. However, Nelson and colleagues [53] systematically identified and quantified decrementally cost-effective health care innovations, for instance, those where the monetary savings had to be large enough to justify the associated QALY loss, i.e., those in the southwest quadrant of the cost-effectiveness plane. To do this they conservatively designated an intervention as decrementally cost-effective if it saved at least $100,000 for each QALY lost. This was based on the existing available data which suggested that people ‘demand’ 2 to 6 fold more per QALY lost than they are willing to ‘pay’ for each QALY [42, 54]. On the basis of this, results from our study would suggest that to move from a universal vaccination strategy to a selective based strategy saves approximately €218,692 per life year lost. Indeed, assuming a willingness to accept threshold of twice the willingness to pay threshold, the probability of such a policy change being cost effective is in the region of 77 %. This finding will be of interest to other health-care systems who are transitioning from a TB high-burden to a TB low-burden society in an era of evidence-based health policy decision making. For policy makers in Ireland, an appropriate next step would be to consider the practical elements of implementing and conducting such a vaccination policy, as successful TB control in low burden countries requires efforts to identify the high risk groups among the population and engage them in screening. This must happen along with rigorous implementation of treatment strategies for cases such as directly observed therapy (DOTs), along with active case finding through contract tracing, to ensure that the health system is able to diagnose and manage cases, regardless of risk factors, as early as possible. Indeed, in order to identify the most optimal and efficient programme to use in the Irish healthcare system, it would be prudent to compare the cost effectiveness of a screening and treatment programme vs a programme of vaccination. However, the current TB control programme would have to be standardised nationally, in order that such an evaluation could make a meaningful contribution to aid the decision maker.

### Ethical considerations

Adopting such a change in vaccination policy also raises some ethical implications. It is likely that there will be an increase in TB cases as a result of changing from universal to selective vaccination in Ireland. It is important therefore that the message is communicated clearly so as to maintain public trust in the vaccination programme. A clearly communicated definition and rationale for classifying high and low risk infants would also be needed in order to target the correct infants and to help gain consent from parents for vaccination of their children.

### Strengths and limitations / Assumptions used

#### Identifying the target group for selective vaccination

For the purposes of this analysis, target group of high risk infants for vaccination were assumed to be those whose families have immigrated from high-incidence countries. In Ireland this is likely to be the largest group of “at risk” infants and data from the Growing Up in Ireland study [7] was used to help quantify this parameter. There will be considerable uncertainty with the figure used here (11.7 % of the birth cohort) as data from some local health offices around the Dublin area showed that for the latter half of 2012 and early 2013, there was considerable variation in the proportion of high risk infants (out of all those vaccinated) brought for BCG vaccination. These figures ranged from 11.8 % in some areas to 47.6 % in other areas (NIO, personal communication). These figures were, however, highly dependent on the clinic location and it is likely that the higher figures will be over estimates. Other age groups not considered in the analysis were children between the ages of one to 16, who were previously unvaccinated, and came from “high risk” families, as were previously unvaccinated under 16 year olds, who were born or lived for prolonged periods in a high endemic area, as it was difficult to quantify these groups. However they are not considered to be the largest “high-risk” group.

### Quality of life

This study did not incorporate a quality of life measurement into the analysis. There is currently a paucity of data in the literature on the quality of life in children, probably due to the absence of an appropriate quality of life instrument. As stipulated in the recent NICE clinical guideline on tuberculosis [55], a study is needed to ascertain quality-of-life score estimates from those with TB (both active disease and latent infection), including adverse treatment effects, using an appropriate quality-of-life instrument. Cost-effectiveness estimates in the form of QALYs would be more comparable with cost-effectiveness estimates from other assessments of vaccines and could assist in improving economic decision-making throughout TB care. However, the incorporation of LYG as the outcome measure does facilitate its comparison with assessments conducted previously by this group (e.g., PCV7 [56], HPV [57], Hepatitis B [58] and rotavirus [59]).

### Uncertainty

The results of the analysis are subject to some uncertainty. One way sensitivity analysis indicates that the ICERs are sensitive to the estimate of vaccine efficacy against deaths. This is in keeping with the evidence from the literature. There is also uncertainty associated with the multiplier which apportions a level of risk to the targeted (i.e., selective) population. In the absence of any definitive evidence on the level of risk in the selective population, the assumption is based on the TB rates in the foreign born population relative to the indigenous population.

## Conclusion

The results of the current study (i) support the protective effect of the BCG vaccine in infants and (ii) suggest that a move from the universal to a selective based strategy could be considered decrementally cost-effective and may offer an opportunity to improve the efficiency of health resource allocation, whereby the resources saved could be applied to specific areas targeted in the national TB control and prevention programme. The results of this study will be of interest to those who are tasked with resource planning and will serve as a beneficial case study for those emerging economies with limited health resources, who are working towards lowering the incidence of TB in their country.

## Abbreviations

BCG, Bacillus Calmette–Guérin; DOTs, directly observed therapy; DRG, diagnosis related group; HPSC, Health Protection Surveillance Centre; HPV, Human Papillomavirus; HSE, Health Service Executive; ICER, incremental cost effectiveness ratio; IUATLD, International Union Against Tuberculosis and Lung Disease; LYG, life year gained; NIAC, National Immunisation Advisory Committee; NIO, National Immunisation Office; PCV, Pneumococcal Conjugate Vaccine; QALY, quality adjusted life year; TB, tuberculosis

## Additional files

Additional file 1: Table S1. Resource utilisation and unit cost data for the direct cost estimate for an episode of latent TB (LTBI). (PDF 93 kb) Additional file 2: Table S2. Resource utilisation and unit cost data for the direct cost estimate for an episode of pulmonary TB. (PDF 113 kb) Additional file 3: Table S3. Resource utilisation and unit cost data for the direct cost estimate for an episode of extrapulmonary TB. (PDF 122 kb) Additional file 4: Table S4. Resource utilisation and unit cost data for the direct cost estimate for an episode of TB Meningitis (PDF 123 kb) Additional file 5: Table S5. Cost of long term complications of meningitis (PDF 109 kb)
