# Supplementary material for: Evaluating the neonatal BCG vaccination programme in Ireland
Source: Arch Public Health. 2016 Jul 13;74:28. doi: 10.1186/s13690-016-0141-0 (PMC4942954; doi:10.1186/s13690-016-0141-0)
Supplement: Additional file 1: Table S1. — Resource utilisation and unit cost data for the direct cost estimate for an episode of latent TB (LTBI). (PDF 93 kb) [file 13690_2016_141_MOESM1_ESM.pdf]

Table 1: Resource utilisation and unit cost data for the direct cost estimate for an episode of latent TB (LTBI).

A. DIAGNOSIS					Quantity (qty)	Unit Cost	Total Cost
Diagnostic Tests							
Tuberculin Skin Test (TST)					1	€20.95	€20.95
Chest X-ray (CXR)					1	€30.00	€30.00
Physician Visits							
Pediatrician Visit					1	€170.93	€170.93
Liver Function Tests (LFTs)					1	€12.80	€12.80
**TOTAL Diagnosis COST**							**€234.68**

B. Treatment	daily dosage	mg/kg	#days	%	qty	Unit Cost	Total Cost
Oral Antibiotics							
Isoniazid	125	5	168	50%	168	€0.79	€66.36
Rifampicin	250	10	168	50%	168	€0.48	€40.32
Follow up Visits							
Pediatrician follow up				50%	1	€170.93	€85.47
Out-patient follow up				50%	1	€139.00	€69.50
GP consult				100%	2	€35.39	€70.78
Clinical Nurse Specialist follow up for management of therapy	5 hrs/week		24weeks	5.00%	24	€272.70	€327.24
**TOTAL (Cost per case of LTBI, detected with mantoux)**							**€894.35**
