# Supplementary material for: Evaluating the neonatal BCG vaccination programme in Ireland
Source: Arch Public Health. 2016 Jul 13;74:28. doi: 10.1186/s13690-016-0141-0 (PMC4942954; doi:10.1186/s13690-016-0141-0)
Supplement: Additional file 2: Table S2. — Resource utilisation and unit cost data for the direct cost estimate for an episode of pulmonary TB. (PDF 113 kb) [file 13690_2016_141_MOESM2_ESM.pdf]

Table 2: Resource utilisation and unit cost data for the direct cost estimate for an episode of pulmonary TB.

**A. DIAGNOSIS**	**Quantity (qty)**	**Unit Cost**	**Total Cost**
Diagnostic Tests			
Tuberculin Skin Test	1	€20.95	€20.95
Chest X-ray (CXR)	1	€30.00	€30.00
Sputum Smear microscopy	3	€58.41	€175.23
Polymerase Chain Reactions (PCRs)	1	€34.92	€34.92
Physician Visits/Hospital days			
Pediatrician Visit	2	€170.93	€341.86
Ophthalmology visit prior to starting ethambutol	1	€170.93	€170.93
Hospital Day (Peds ward)	9	€555.00	€4,995.00
**TOTAL Diagnosis COST**			**€5,768.89**

**B. Initial Treatment success**	**daily dosage (mg/kg)**		**#days**	**%**	**qty**	**Unit Cost**	**Total Cost**
Oral Antibiotics							
Isoniazid	125	5	168	100%	168	€0.79	€132.72
Rifampicin	250	10	168	100%	168	€0.48	€80.64
Pyrazinamide	625	25	56	100%	56	€0.39	€21.84
Ethambutol	500	20	56	100%	56	€2.10	€117.79
Physician Visits/Hospital Days							
Follow-up Pediatrician visits					6	€170.93	€1,025.58
Liver Function Tests (LFTs)					6	€12.80	€76.80
Sputum smears & cultures					3	€58.41	€175.23
Full Blood Count (FBC)					6	€16.00	€96.00

B. Initial Treatment success (cont)		daily dosage (mg/kg)	#days	%	qty	Unit Cost	Total Cost
Follow-up							
Follow-up CXR after 1 mth					1	€30.00	€30.00
Repeat CXR					1	€30.00	€30.00
Eye review ophthalmology					1	€170.93	€170.93
Management of therapy							
Clinical Nurse Specialist	5 hrs/week	24weeks		5.00%	24	€272.70	€327.24
**TOTAL Treatment Success COST**							**€2,284.77**

€8,053.66

C. Treatment Failure	daily dosage (mg/kg)		#days	%	qty	Unit Cost	Total Cost
Oral Antibiotics							
Isoniazid	125	5	168	100%	56	€0.79	€44.24
Rifampicin	250	10	168	100%	56	€0.48	€26.88
Pyrazinamide	625	25	56	100%	56	€0.39	€21.84
Ethambutol	500	20	56	100%	56	€2.10	€117.79
Physician Visits/Hospital Days							
Sputum smears & cultures					1	€58.41	€58.41
Follow-up Pediatrician visits					1	€170.93	€170.93
**TOTAL Treatment Failure COST**							**€440.09**

D. Revised Treatment regimen success	daily dosage (mg/kg)		#days	%	qty	Unit Cost	Total Cost
Oral Antibiotics							
Isoniazid	125	5	168	100%	168	€0.79	€132.72
Rifampicin	250	10	168	100%	168	€0.48	€80.64
Pyrazinamide	625	25	28	100%	28	€0.39	€10.92
Ethambutol	500	20	168	100%	168	€2.10	€353.37

D. Revised Treatment regimen success	daily dosage (mg/kg)	#days	%	qty	Unit Cost	Total Cost
Physician Visits/Hospital Days						
Follow-up Pediatrician visits				6	€170.93	€1,025.58
LFTs				6	€12.80	€76.80
Sputum smears & cultures				3	€58.41	€175.23
FBC				6	€16.00	€96.00
Follow-up						
Follow-up CXR after 1 mth				1	€30.00	€30.00
Repeat CXR				1	€30.00	€30.00
Eye review ophthalmology				1	€170.93	€170.93
Management of therapy						
Clinical Nurse Specialist	5 hrs/week	24weeks	5.00%	24	€272.70	€327.24
**TOTAL Treatment Success COST**						**€2,509.43**
