# Supplementary material for: Evaluating the neonatal BCG vaccination programme in Ireland
Source: Arch Public Health. 2016 Jul 13;74:28. doi: 10.1186/s13690-016-0141-0 (PMC4942954; doi:10.1186/s13690-016-0141-0)
Supplement: Additional file 3: Table S3. — Resource utilisation and unit cost data for the direct cost estimate for an episode of extrapulmonary TB. (PDF 122 kb) [file 13690_2016_141_MOESM3_ESM.pdf]

Table 3: Resource utilisation and unit cost data for the direct cost estimate for an episode of extrapulmonary TB.

A. DIAGNOSIS	No of days	%	Quantity (qty)	Unit Cost	Total Cost
Diagnostic Tests					
Tuberculin Skin Test		100.0%	1	€20.95	€20.95
Chest X-ray (CXR)		100.0%	1	€30.00	€30.00
Sputum Smear microscopy		100.0%	3	€58.41	€175.23
IGRA		50.0%	1	€65.00	€32.50
CT Scan		68.9%	1	€110.00	€75.79
MRI		10.0%	1	€194.00	€19.40
Ultrasound		36.8%	1	€115.00	€42.32
Peritoneal biopsy		4.7%	1	€ 475.00	€22.33
Lymph node biopsy (INTRA-Thoracic)		9.5%	1	€ 4,389.21	€416.97
Lymph node biopsy (EXTRA-Thoracic)		26.8%	1	€ 745.81	€199.88
Liver biopsy (needle)		3.7%	1	€ 948.00	€35.08
Bone Biopsy		3.2%	1	€ 291.00	€9.31
Pleural biopsy		23.7%	1	€ 922.00	€218.51
Other (Bronchoscopy)		15.8%	1	€ 478.00	€75.52
Genitourinary (D&C)		0.5%	1	€ 399.00	€2.00
LP/CSF (incl C&S)		11.0%	1	€ 158.30	€17.41
PCRs		100.0%	1	€ 34.92	€34.92
Unknown		1.0%	1	€ 347.00	€3.47
Physician Visits/Hospital days					
Pediatrician consult				€ 170.93	€ 170.93
ID/micro consult				€ 170.93	€ 170.93
Hospital Day (Peds ward)	14			€555	€ 7,770.00
**TOTAL Diagnosis COST**					**€ 9,543.45**

B. Treatment Success		daily dosage (mg/kg)		#days	%	Quantity (qty)	Unit Cost	Total Cost
**Medical**								
Oral Antibiotics								
Isoniazid		125	5	168	100.0%	168	€0.79	€132.72
Rifampicin		250	10	168	100.0%	168	€0.48	€80.64
Pyrazinamide		625	25	56	100.0%	56	€0.39	€21.84
Ethambutol		500	20	56	100.0%	56	€2.10	€117.79
**Surgical**								
Anterior spinal fusion					3.2%		€ 2,973.00	€ 95.14
Physician Visits/Hospital Days								
Follow-up Pediatrician visits						6	€ 170.93	€ 1,025.58
Liver Function Tests (LFTs)						6	€ 12.80	€ 76.80
CT Scan					68.9%	2	€110.00	€151.58
MRI					10.0%	2	€194.00	€38.80
Ultrasound					36.8%	2	€115.00	€84.64
Full Blood Count (FBC)						9	€ 16.00	€144.00
Follow-up								
Opthalmology						1	€ 170.93	€ 170.93
Management of therapy								
Clinical Nurse Specialist	5 hrs/week		24weeks		5.00%	24	€272.70	€327.24
**TOTAL Treatment Success COST**								**€ 2,467.70**

C. Treatment Failure	daily dosage (mg/kg)		#days	%	Quantity (qty)	Unit Cost	Total Cost
**Medical**							
Oral Antibiotics							
Isoniazid	125	5	168	100.0%	56	€0.79	€44.24
Rifampicin	250	10	168	100.0%	56	€0.48	€26.88
Pyrazinamide	625	25	56	100.0%	56	€0.39	€21.84
Ethambutol	500	20	56	100.0%	56	€2.10	€117.79
Physician Visits/Hospital Days							
Follow-up Pediatrician visits					1	€ 170.93	€ 170.93
Sputum smears & cultures					1	€58.41	€58.41
**TOTAL Treatment Failure COST**							**€ 440.09**

D. Revised Treatment Success	daily dosage (mg/kg)		#days	%	Quantity (qty)	Unit Cost	Total Cost
**Medical**							
Oral Antibiotics							
Isoniazid	125	5	168	100.0%	280	€0.79	€221.20
Rifampicin	250	10	168	100.0%	280	€0.48	€134.40
Pyrazinamide	625	25	56	100.0%	56	€0.39	€21.84
**Surgical**							
Anterior spinal fusion				3.2%		€ 2,973.00	€ 95.14
Physician Visits/Hospital Days							
Follow-up Pediatrician visits					10	€ 170.93	€ 1,709.30
LFTs					10	€ 12.80	€ 128.00
CT Scan				68.9%	2	€110.00	€151.58

D. Revised Treatment Success		daily dosage (mg/kg)	#days	%	Quantity (qty)	Unit Cost	Total Cost
MRI				10.0%	2	€194.00	€38.80
Ultrasound				36.8%	2	€115.00	€84.64
FBC					9	€ 16.00	€144.00
Follow-up							
Opthalmology					1	€ 170.93	€ 170.93
Management of therapy							
Clinical Nurse Specialist	5 hrs/week	40weeks		5.00%	40	€272.70	€545.40
**TOTAL Treatment Success COST**							**€ 3,445.23**
