# Supplementary material for: Evaluating the neonatal BCG vaccination programme in Ireland
Source: Arch Public Health. 2016 Jul 13;74:28. doi: 10.1186/s13690-016-0141-0 (PMC4942954; doi:10.1186/s13690-016-0141-0)
Supplement: Additional file 4: Table S4. — Resource utilisation and unit cost data for the direct cost estimate for an episode of TB Meningitis (PDF 123 kb) [file 13690_2016_141_MOESM4_ESM.pdf]

Table 4: Resource utilisation and unit cost data for the direct cost estimate for an episode of TB Meningitis.

1. Diagnosis			Probability (%)	Quantity (qty)	Unit Cost	Total Cost
Physician Visits/Hospital Days						
Pediatric ER visit			100%	1.5	€ 222.00	€ 333.00
**TOTAL**						**€ 333.00**
Diagnostic Tests						
LP/CSF analysis (incl.C+S)			100%	1	€ 158.00	€ 158.00
Blood C+S			100%	1	€ 29.10	€ 29.10
Urine C+S			100%	1	€ 29.10	€ 29.10
FBC			100%	1	€ 16.00	€ 16.00
IGRA			100.0%	1	€65.00	€65.00
CXR			65%	1	€ 30.00	€ 19.50
CT head			30%	1	€ 96.00	€ 28.80
EEG			10%	1	€ 174.60	€ 17.46
PCRs			100%	1	€ 34.92	€ 34.92
**TOTAL**						**€ 397.88**
Physician Visits						
Pediatrician consult					€ 170.93	€ 170.93
ID/micro consult					€ 170.93	€ 170.93
**TOTAL**						**€ 341.86**
Antibiotics	**daily dosage (mg/kg)**	**#days**				
Isoniazid	5	365	100%	365	€0.79	€288.35
Rifampicin	10	365	100%	365	€0.48	€175.20
Pyrazinamide	25	365	100%	365	€0.39	€142.35
Ethambutol	20	365	100%	365	€2.10	€767.74
Prednisolone	1	365	100%	365	0.14	€51.10
Management of therapy						
Clinical Nurse Specialist	5 hrs/we		5%	52 weeks	€ 272.70	€ 709.02
**TOTAL**						**€ 2,133.76**
**Diagnosis Total**						**€ 3,206.50**

2. Stable patient	Probability (%)	Quantity	Unit Cost	Total Cost
Physician Visits/Hospital Days				
Hospital stay (Peds ward)	100%	14	€ 555.00	€ 7,770.00
**TOTAL**				€ 7,770.00
**Stable patient Total**				**€ 7,770.00**

3. Success (medical)	Probability (%)	Quantity	Unit Cost	Total Cost
Physician Visits/Hospital Days				
Follow-up Pediatrician visit	100%	3	€ 170.93	€ 512.79
**TOTAL**				€ 512.79
**Success (medical) Total**				**€ 512.79**

4. Persistent Fever	Probability (%)	Quantity	Unit Cost	Total Cost
Physician Visits/Hospital Days				
Hospital Day (Peds ward)	100%	8	€ 555.00	€ 4,440.00
**TOTAL**				**€ 4,440.00**
Diagnostic Tests				
LP/CSF analysis (incl.C+S)	50%	1	€ 158.00	€ 79.00
Blood C+S	100%	1	€ 29.10	€ 29.10
FBC	100%	1	€ 16.00	€ 16.00
CXR	60%	1	€ 30.00	€ 18.00
CT head	70%	1	€ 96.00	€ 67.20
MRI head	10%	1	€ 190.00	€ 19.00
EEG	10%	1	€ 174.60	€ 17.46
**TOTAL**				**€ 245.76**
**TOTAL**				**€ 4,685.76**
**Persistent Fever Total**				**€ 4,685.76**

5. Respond to treatment	Probability (%)	Quantity	Unit Cost	Total Cost
Physician Visits/Hospital Days				
Pediatric Neurosurgical Consult	100%	1	€ 170.93	€ 170.93
**TOTAL**				**€ 170.93**
**Respond to treatment Total**				**€ 170.93**

6. Septic Patient	Probability (%)	Quantity	Unit Cost	Total Cost
Physician Visits/Hospital Days				
ICU Day	100%	5	€ 2,051.40	€ 10,257.00
Hospital days	100%	10	€ 555.00	€ 5,550.00
**TOTAL**				**€ 15,807.00**
Diagnostic Tests				
Co ag test		1	€ 60.53	€ 60.53
LP/CSF analysis (incl.C+S)	75%	1	€ 158.30	€ 118.73
FBC	100%	4	€ 16.00	€ 64.00
renal profile	100%	1	€ 10.00	€ 10.00
LFTs	100%	1	€ 12.80	€ 12.80
MRI	100%	1	€ 194.00	€ 194.00
**TOTAL**				**€ 460.06**
				**€ 0.00**
**Septic Patient total**				**€ 16,267.06**

7. Neurosurgical Drainage	Probability (%)	Quantity	Unit Cost	Total Cost
Physician Visits/Hospital Days				
Neurosurgical Drainage of	100%	1	€ 3,430.64	**€ 3,430.64**
Hospital days	100%	7	€ 555.00	**€ 3,885.00**
**TOTAL**				**€ 7,315.64**
**Neurosurgical Drainage Total**				**€ 7,315.64**

8. Success (surgical)	Probability (%)	Quantity	Unit Cost	Total Cost
Physician Visits/Hospital Days				
Follow-up Pediatrician visit	100%	9	€ 170.93	€ 1,538.37
Follow-up Pediatric Neurosurgeon	100%	3	€ 170.93	€ 512.79
**TOTAL**				**€ 2,051.16**
**Success (surgical) Total**				**€ 2,051.16**

10. Spontaneous resolution	Probability (%)	Quantity	Unit Cost	Total Cost
Physician Visits/Hospital Days				
Follow-up Pediatrician visit	100%	9	€ 170.93	€ 1,538.37
Pediatric Neurosurgical Consult	100%	1	€ 170.93	€ 170.93
**TOTAL**				**€ 1,709.30**
**Spontaneous resolution total**				**€ 1,709.30**

9. Failure	Probability (%)	Quantity	Unit Cost	Total Cost
Physician Visits/Hospital Days				
Hospital Day (Peds ward)	100%	6	€ 555.00	€ 3,330.00
**TOTAL**				**€ 3,330.00**
**TOTAL**				**€ 0.00**
**Failure/Death Total**				**€ 3,330.00**
