# Supplementary material for: Evaluating the neonatal BCG vaccination programme in Ireland
Source: Arch Public Health. 2016 Jul 13;74:28. doi: 10.1186/s13690-016-0141-0 (PMC4942954; doi:10.1186/s13690-016-0141-0)
Supplement: Additional file 5: Table S5. — Cost of long term complications of meningitis (PDF 109 kb) [file 13690_2016_141_MOESM5_ESM.pdf]

Table 5: Cost of long term complications of meningitis

HEARING LOSS	Probability (%)	Quantity	Unit Cost	Total Cost
Health Care Professional Visits				
Follow-up Pediatrician visit	100%	3	€170.93	€ 512.79
Speech Therapist	50%	3	€ 54.96	€ 82.44
**TOTAL**				**€ 595.23**
Diagnostic Tests				
Audiometry	100%	3	€ 30.13	€ 90.39
ABR/BAER	100%	2	€ 66.17	€ 132.34
**TOTAL**				**€ 222.73**
Non-Surgical Therapies				
Hearing Aids (2)	90%	1	€ 3,057.92	€ 2,752.13
**TOTAL**				**€ 2,752.13**
Surgical Procedures				
Cochlear Implantation	0.5%	1	€ 51,707.00	€ 258.54
**TOTAL**				**€ 258.54**
**HEARING LOSS: TOTAL COST**				**€ 3,828.62**

DEV. DELAY/MR	Probability (%)	Quantity	Unit Cost	Total Cost
Health Care Professional Visits				
Follow-up Pediatrician visit	100%	6	€170.93	€ 1,025.58
Occupational Therapist	100%	6	€ 54.96	€ 329.76
Physiotherapist	100%	12	€ 54.96	€ 659.52
Speech Therapist	100%	12	€ 54.96	€ 659.52
**TOTAL**				**€ 2,674.38**
Diagnostic Tests				
Psychometric Testing	100%	1	€ 953.42	€ 953.42
**TOTAL**				**€ 953.42**
**DEV. DELAY/MR: TOTAL COST**				**€ 3,627.80**

FOCAL NEURO DEFICITS	Probability (%)	Quantity	Unit Cost	Total Cost
Health Care Professional Visits				
Pediatric Neurologist	100%	3	€ 170.93	€ 512.79
Pediatric Neurosurgeon(initial visit)	40%	1	€ 170.93	€ 68.37
Pediatric Neurosurgeon	40%	1	€ 170.93	€ 68.37
Pediatric Orthopedic Surgeon (initial visit)	25%	1	€ 170.93	€ 42.73
Occupational Therapist	100%	6	€ 54.96	€ 329.76
Physiotherapist	100%	12	€ 54.96	€ 659.52
Speech Therapist	100%	12	€ 54.96	€ 659.52
**TOTAL**				**€ 2,341.07**
Diagnostic Tests				
CT head	100%	1	€ 96.00	€ 96.00
EEG	100%	1	€ 174.60	€ 174.60
**TOTAL**				**€ 270.60**
Surgical Procedures				
Ventriculoperitoneal Shunt Placement/Revision	20%	1	€ 3,430.64	€ 686.13
**TOTAL**				**€ 686.13**
**FOCAL NEURO DEFICITS: TOTAL COST**				**€ 3,297.79**

CHRONIC SEIZURES	Probability (%)	Quantity	Unit Cost	Total Cost
Health Care Professional Visits				
Follow-up Pediatrician visit			170.93	€170.93
Pediatric Neurologist	100%	3	€ 170.93	€ 512.79
**TOTAL**				**€ 512.79**
Diagnostic Tests				
EEG	100%	2	€ 174.60	€ 349.20
**TOTAL**				**€ 349.20**
Non-Surgical Therapies				
Carbamazepine (400mg)	100%	365	€ 0.16	€ 58.40
**TOTAL**				**€ 58.40**
**CHRONIC SEIZURES: TOTAL COST**				**€ 1,091.32**

Table 6: Long term cost of disability per patient over 15 years

Long term cost per disability (cost per patient over 15 years)		Likelihood of disability	Cost * Probability
Hearing Loss	€46,396.74	7.0%	€3,247.77
Dev. Delay	€43,963.05	50.0%	€21,981.53
Focal Neuro Deficits	€39,963.96	25.0%	€9,990.99
Chronic Seizures	€13,225.04	7.6%	€1,005.10
**Long-term cost of disability in € per case (over 15 yrs)**			**€36,225.39**
